# Factors associated with poorer quality of life in people living with HIV in southwestern France in 2018–2020 (ANRS CO3 AQUIVIH-NA cohort: QuAliV study)

**DOI:** 10.1038/s41598-023-43434-x

**Published:** 2023-10-02

**Authors:** Diana Barger, Mojgan Hessamfar, Didier Neau, Sophie Farbos, Olivier Leleux, Charles Cazanave, Nicolas Rouanes, Pierre Duffau, Estibaliz Lazaro, Patrick Rispal, François Dabis, Linda Wittkop, Fabrice Bonnet

**Affiliations:** 1 Univ. Bordeaux, INSERM, BPH, U1219, 33000 Bordeaux, France; 2 CHU de Bordeaux, Service de Médecine Interne et Maladies Infectieuses, Hôpital Saint-André, 33000 Bordeaux, France; 3 CHU de Bordeaux, COREVIH Nouvelle Aquitaine, INSERM, U1219, 33000 Bordeaux, France; 4 CHU de Bordeaux, Service des Maladies Infectieuses et Tropicales, INSERM, U1219, 33000 Bordeaux, France; 5 CH de la Côte Basque, Service de Maladies Infectieuses, 64000 Bayonne, France; 6 Univ. Bordeaux, INSERM, Institut Bergonié, BPH, U1219, CIC-EC 1401, 33000 Bordeaux, France; 7 CH de Périgueux, Service de Médecine Polyvalente, 24019 Périgueux, France; 8 Univ. Bordeaux, Department of Immunology, CNRS, ImmunoConcEpT, UMR 5164, 33000 Bordeaux, France; 9 CHU de Bordeaux, Service de Médecine Interne, 33604 Pessac, France; 10 CH de Agen-Nerac, Service de Médecine Interne, 47923 Agen, France; 11 INRIA, SISTEM Team, 33400 Talence, France; 12 CHU de Bordeaux, Service d’information médicale, INSERM, Institut Bergonié, CIC-EC 1401, 33000 Bordeaux, France

**Keywords:** Public health, Quality of life, Viral infection

## Abstract

We evaluated people living with Human Immunodeficiency Virus’ (PLWH) quality of life (QoL) and assessed whether their demographic, disease-related, socioeconomic, or behavioral characteristics were associated with poorer QoL. ANRS CO3 AQUIVIH-NA cohort participants (Nouvelle Aquitaine, France) were recruited to a cross-sectional study (2018–2020) and their QoL assessed (WHOQOL-BREF). We calculated median (Q1, Q3) QoL domain scores and assessed factors associated with poorer median QoL using bivariable and multivariable quartile regression. Of the 965 PLWH included, 98.4% were on antiretroviral therapy, 94.7% were virally-suppressed, 63.5% reported good/very good QoL. Median scores (0–100) were highest for physical (69;Q1, Q3: 56, 81) and environmental (69; 56, 75) QoL and lowest for social (56; 44, 69) and psychological (56; 44, 69) QoL. PLWH with ≥ 3 comorbidities, HIV-related stigma, or income of < 1500€/month had poorer median adjusted physical, psychological, social, and environmental QoL scores compared to reference groups. While more than half of PLWH reported good/very good QoL, we have not achieved good QoL in 90% of PLWH. Multi-morbidity, HIV-related stigma, and social determinants were consistently and independently associated with poorer QoL. Addressing structural factors in addition to those indirectly related to HIV is required to attain good QoL in all PLWH.

## Introduction

Early and sustained access to antiretroviral therapy (ART) for those diagnosed with Human Immunodeficiency Virus (HIV), irrespective of CD4 cell count, has been the standard of care in France since 2013^1^. The evolving needs of people living with HIV(PLWH) have prompted calls to “go beyond viral suppression” or to ensure good [health-related] quality of life (QoL) at all stages of the HIV care continuum. In reference to the Joint United Nations Programme on HIV/AIDS’ (UNAIDS) 90–90-90 targets^2^, this concept has been termed the 4th 90^3,4^. The World Health Organisation (WHO) defines QoL as “an individual’s perception of their position in life in the context of the culture and value systems in which they live and in relation to their goals, expectations, standards and concerns”^5^. Seen as too nebulous, the term *health-related* QoL (HRQoL) emerged, namely to meet the needs of clinical medicine/trials. HRQoL reflects “the patient’s perception of the effect of illness and treatment on physical, psychological, and social aspects of life”^6^. Early research on HRQoL in PLWH confirmed that disease status and severity resulted in symptoms associated with poorer functional status and HRQoL^7^. It has since been suggested that socio-demographic, psychological, clinical and behaviour characteristics are also associated with HRQoL and its specific domains^8^. However, the direction of these associations and/or their significance is unclear. Findings from studies comparing the HRQoL of treated and mostly virally suppressed PLWH in high-income settings to that of their general population counterparts have been discordant, with some concluding that PLWH’s HRQoL is persistently poorer after adjusting for potential confounders^9^ or, more recently, that their HRQoL is comparable^10^.

We assessed overall QoL and physical, psychological, social, and environmental QoL in PLWH in Nouvelle Aquitaine, France. We then investigated whether PLWH’s demographic/epidemiological, disease-related, socio-economic, and/or behavioral characteristics of PLWH were associated with poorer QoL.

## Materials and methods

### Study design

The ANRS CO3 AQUIVIH-NA cohort is an open, prospective hospital-based cohort of HIV-1 positive adults ($\ge$ 18 years old) from 15 hospitals in Nouvelle Aquitaine, France. Clinical Research Associates collected and standardized clinical and laboratory data from participants’ medical records via an electronic Case Report Form. The QuAliV study is a multi-centric cross-sectional study conducted within the cohort to assess QoL and other patient-reported outcomes^11^. Investigators recruited cohort participants at routine hospital-based HIV consultations between the 23/07/2018 and 31/12/2019 in Bordeaux, Bayonne, Périgueux, and Agen in Nouvelle Aquitaine, France. Participants had to complete a web-based or pen-and-paper assessment independently following their HIV-consultation^11^.

### Measures

Sociological and behavioral variables were self-reported via the QuAliV assessment, whereas demographic, epidemiological, clinical, and laboratory data were collected via the cohort information system. [HR]QoL was collected using the French version of the disease-specific WHOQOL-HIV BREF instrument, which is derived from the generic 26-item World Health Organization’s Quality Of Life Instrument, named WHOQOL-BREF, and the HIV-specific 100-item WHOQOL-HIV^12^. We analysed the 26-item WHOQOL-BREF covering four domains: physical, psychological, social, and environmental QoL and two general items measuring overall QoL and general health satisfaction (GHS). The WHOQOL-BREF embodies the WHO’s definition of QoL as multi-dimensional in nature and its measure subjective, embedded in a cultural, social and environmental context. Items were coded on a scale from 1 (worst) to 5 (best).

Demographic and epidemiological variables included age, birth place, and a composite variable comprising sex assigned at birth and mode of HIV acquisition (men who have sex with men [MSM], men who acquired HIV via heterosexual contact/other, women who acquired HIV via heterosexual contact/other, men who inject(ed) drugs, and women who inject(ed) drugs). Disease-related variables included duration of HIV infection, Centers for Disease Control and Prevention (CDC) clinical categories for HIV, CD4 cell count (mm^3^) and viral load (copies/mL) recorded within a ±2 year window of the QuAliV assessment, number of comorbidities (chronic renal failure, history of cardiovascular events, hypertension [taking antihypertensive treatment], diabetes, and cancer), history of Hepatitis Virus C (HCV) co-infection, and perceived HIV-related stigma^13^. Perceived HIV-stigma was evaluated with the question: “To what extent are you bothered by people blaming you for your HIV status?” If participants responded “not at all”, “a little”/ “a moderate amount”, or “very much”/ “an extreme amount”, we assumed that they experienced “none”, “moderate” or “severe” HIV-stigma respectively. Participants reported educational attainment, monthly household income, partnership status, and whether or not they currently smoked.

### Study size

We considered 965 participants who had completed item 1 of the QoL assessment as of March 31, 2020, coinciding with the beginning of the Coronavirus disease 2019 pandemic restrictions.

### Statistical methods

Data were analyzed using STATA 15.1. We analyzed unit non response (e.g. refusal, self-selection), item non response (e.g. skipped questions) and explored reasons for missingness. We compared participants’ demographic/epidemiological characteristics to those of participants actively followed up at the same hospitals within the ANRS AQUIVIH-NA Cohort. Frequencies and proportions were calculated for categorical variables and medians and the 1st and 3rd quartiles for continuous variables. WHOQOL-BREF domain scores were calculated as per guidance. If no more than one item was missing from the physical or environmental health domains, we imputed a horizontal mean based on responses of remaining items. If more than one item was missing, the domain score was not calculated. Similarly, if any items were missing from the psychological and social domains, a domain score was not calculated for the participant. Scores from 4 to 20 were generated which were transformed to scores ranging from 0 to 100 (a higher score reflecting better QoL)^14^. Median domain scores were calculated by demographic/epidemiological, disease-related, socio-economic, and behavioral characteristics as transforme QoL scores were negatively skewed. We tested the null hypothesis of equal median domain scores across groups using the Kruskal–Wallis equality-of-populations rank test, retaining variables with a *p* < 0.02. We fitted successive bivariable and multivariable quantile regression models to assess the effects of demographic/epidemiological, disease-related, social/economic, and behavioral factors on physical, psychological, social, and environmental QoL domain scores. Multivariable models were adjusted for age, sex–HIV acquisition mode (reference [ref.] heterosexual/other men), birth place (ref. natives), HIV infection duration (ref. 2–5 years), clinical disease stage (ref. Stage A), number of comorbidities (ref. 0), history of HCV co-infection (ref. never), perceived HIV-related stigma (ref. none), highest qualification (ref. secondary education), net monthly household income (ref. > 1500€/month), partnership (ref. partnered), and smoking status (ref. non-smoker). We estimated conditional median of QoL domain scores and reported β coefficients and 95% confidence intervals, which reflect the average difference in median QoL scores for the category in question compared to the reference category. We considered *p* values ≤ 0.05 to be indicative of statistical significance. We present the results of the bivariable and multivariable models jointly in regression coefficient plots for each domain. Negative values indicate lower median scores (poorer QoL), whereas positive values indicate better median scores (better QoL).

### Compliance with ethical standards

The ANRS CO3 Aquitaine-AQUIVIH-NA cohort study was approved by an Institutional Review Board (Comité de Protection de Personnes Sud-Ouest et Outre-Mer III) on May 27, 2016 and the QuAliV study was granted ethical approval in August 2017. The National Commission on Informatics and Liberty (CNIL), the French regulatory agency charged with enforcing data privacy laws, reviewed and approved QuAliV study-specific amendments to authorizations on March 12, 2018. Written and informed consent is required of all cohort participants. All methods were carried out in accordance with relevant guidelines and regulations.

## Results

### Sample characteristics

2514 individuals in HIV care were approached prior to 31/12/2019, of whom 2218 (88.2%) were eligible and willing to participate; 1061 (47.8% [1061/2218]) registered and completed the web-based assessment (n = 727) or returned a paper questionnaire (n = 334). We considered data from 965 participants for whom we calculated domain scores for 951 (physical), 918 (psychological), 924 (social), and 944 (environmental). Our sample comprised 726 (75.2%) men and 239 (24.8%) women diagnosed for a median of 19.7 years (Q1, Q3:11.9, 28.0). Nearly all participants (98.4%, [950/965]) were currently on ART, and 94.7% [850/898] had achieved viral suppression. Fifty-five percent had at least one comorbidity, 19.0% had a history of HCV co-infection, and one-third reported experiencing either moderate or severe HIV-related stigma (Table 1). Our analytic sample’s clinical characteristics were comparable to those included in the wider ANRS CO3 AQUIVIH-NA Cohort (Supplementary Material I).

**Table 1:** Sample characteristics (N = 965).

Characteristics	Categories	N (%) or median (Q1, Q3)
Median age, years		55.3 (48.4, 62.0)
Sex-acquisition mode	MSM	481 (49.8)
	Hetero/other men	179 (18.6)
	Hetero/other women	203 (21.0)
	Men who inject(ed) drugs	66 (6.8)
	Women who inject(ed) drugs	36 (3.4)
% France-born		832 (86.2)
Duration of HIV infection, years	0–2	41 (4.3)
	> 2–5	54 (5.6)
	> 5–15	251 (26.0)
	> 15	619 (64.2)
% CD4 cell count ≧ 500 cells/m3^1^		666 (69.0)*
% Viral load < 50 copies/ml^1^		858 (88.9)*
N of comorbidities^2^	0	438 (45.4)
	1	231 (23.9)
	2	145 (15.0)
	3	104 (10.8)
	Unknown	47 (4.9)
Hepatitis C Virus (HCV) co-infection		182 (19.0)
	Unknown	30 (3.1)
HIV-related stigma	None	640 (66.3)
	Moderate	191 (19.8)
	Severe	121 (12.5)
Educational attainment	Primary or less	139 (14.4)
	Secondary	472 (50.7)
	Higher	337 (34.9)
% Income ≥ 1500€/month		551 (57.7)
% Partnered		434 (45.0)
% Never smoked		281 (29.1)*

Key 1: 1 Last measure recorded within 2 years of the QuAliV assessment. 2 Chronic renal failure, history of cardiovascular events, hypertension [taking an antihypertensive treatment], diabetes, and cancer. Diagnoses and prescribed treatments recorded using standard national and international nomenclature (International Disease Classification-10, Unités Communes de Dispensation, ATD). * Denominators taking into account missing data.

### Description of overall perceived quality of life, general health satisfaction, and specific domain scores

Among the 965 participants, 13 (1.4%) rated their QoL as very poor, 51 (5.3%) as poor, 288 (29.8%) as neither good nor poor, 477 (49.4%) as good, and 136 (14.1%) as very good. Twenty-eight participants (2.9%) were very dissatisfied with their health, 126 (13.1%) dissatisfied, 202 (20.9%) neither satisfied nor dissatisfied, 495 (51.3%) satisfied, and 114 (11.8%) very satisfied. The overall median domain scores were highest for physical and environmental QoL, estimated at 69 (Q1, Q3:56, 81) and 69 (56, 75), respectively, and lowest for social and psychological QoL, estimated at 56 (50, 75) and 56 (44, 69), respectively. Overall, women and men who inject(ed) drugs had significantly poorer physical, psychological, social, and environmental QoL. While MSM, heterosexual/other men and women had similar median physical and environmental QoL, MSM and hetero/other women had significantly poorer psychological QoL compared to heterosexual/other men.

### Factors associated with poorer physical, psychological, social and environmental QoL

**Figure 1:**
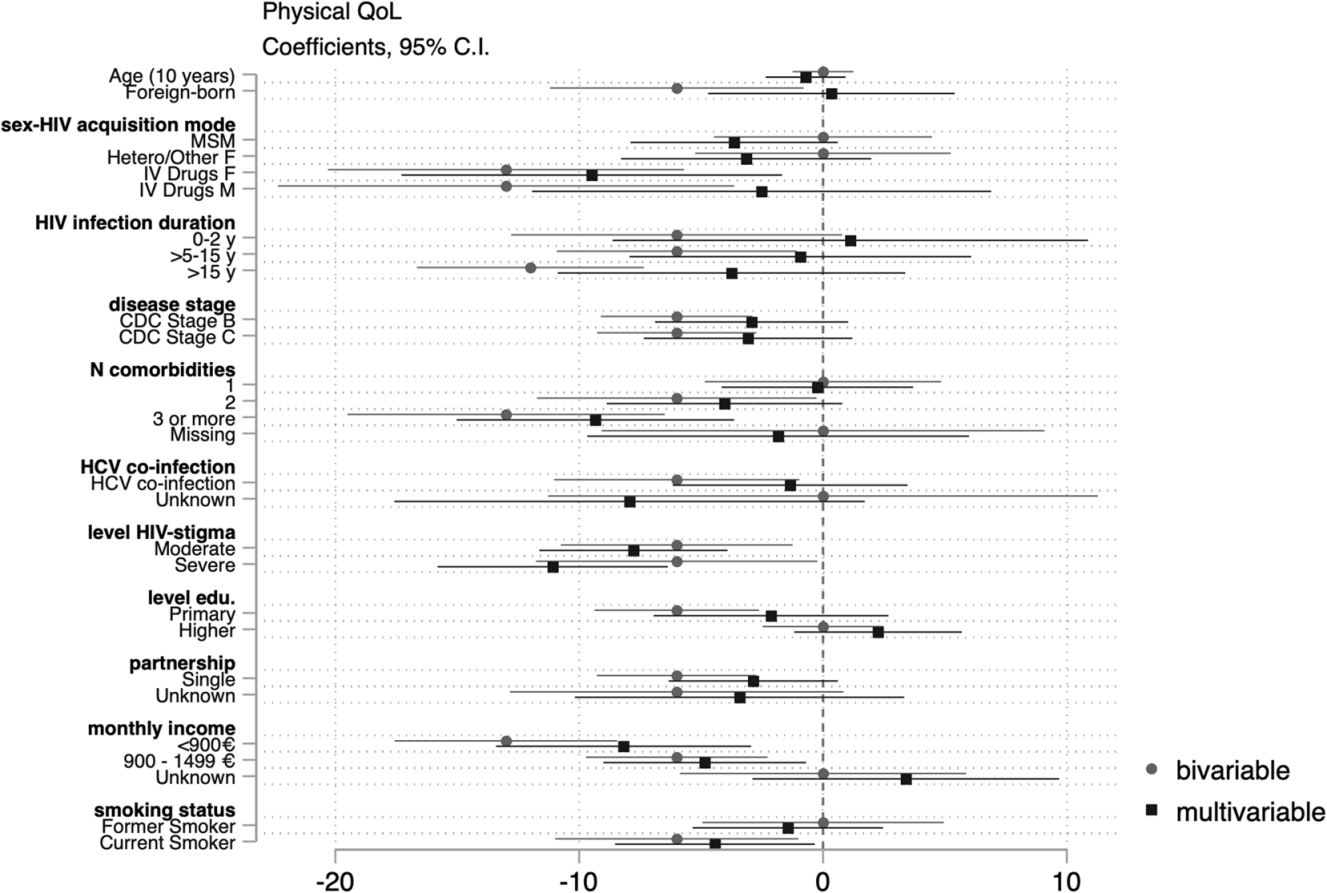
Coefficient plot: Bivariable and multivariable quartile regression analysis of physical QoL.

Those who were foreign-born, had acquired HIV via drug use (men and women), had been living with HIV for > 5 years, had experienced symptomatic HIV or AIDS, had >2 comorbidities, had a history of HCV co-infection, moderate or severe HIV-related stigma, were single, had lower net monthly income, or were smokers had significantly lower physical QoL scores compared to the reference categories in the bivariable analyses. In the multivariable model, having more than three comorbidities, perceiving moderate orsevere HIV-related stigma, earning < 1500€/month, and smoking were independently associated with lower median physical QoL scores (Fig. 1).

**Figure 2:**
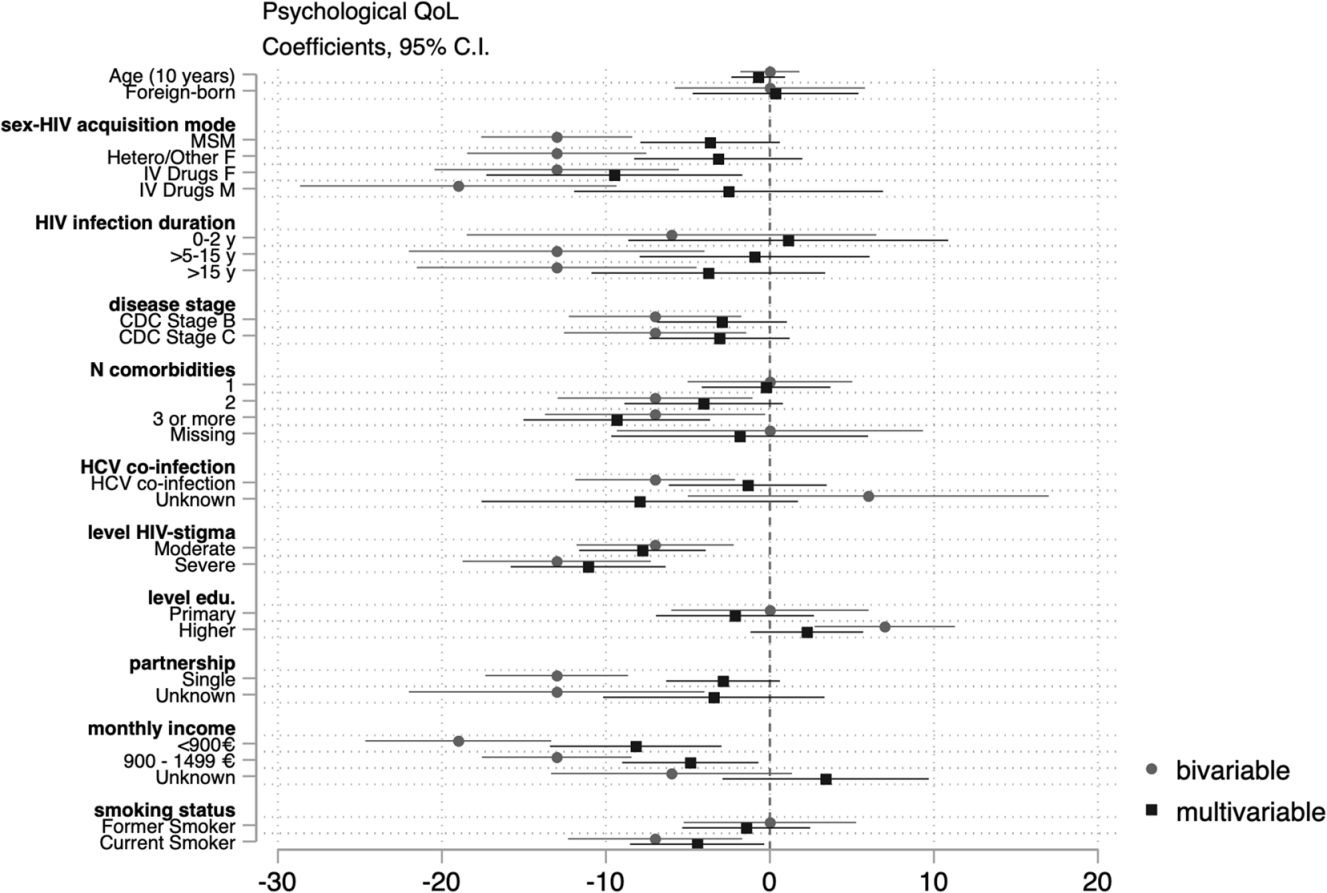
Coefficient plot: Bivariable and multivariable quartile regression analysis of psychological QoL.

MSM, heterosexual/other women, and women and men who inject(ed) IV drugs reported significantly poorer psychological QoL scores than heterosexual/other men. Those who had been diagnosed with HIV for more than five years, had experienced symptomatic HIV or AIDS, had more than two comorbidities, reported moderate or severe HIV-related stigma, were single, had lower net monthly income, or were smokers had significantly lower psychological QoL scores compared to the reference categories in bivariable analyses. However, unlike physical QoL, we found no differences in the median psychological QoL between foreign-born and native PLWH. In the multivariable analysis, those who reported moderate or severe HIV-related stigma, had more than two comorbidities, earned < 1500€/month, and were single had significantly lower psychological QoL scores (Fig. 2). In adjusted analyses, MSM, heterosexual/other women, and women who inject(ed) drugs continued to have significantly lower psychological QoL scores than heterosexual men. However, crude differences in psychological QoL between men who inject(ed) drugs and heterosexual/other men were confounded by disease-related, socioeconomic, and behavioral factors (Fig. 2).

**Figure 3:**
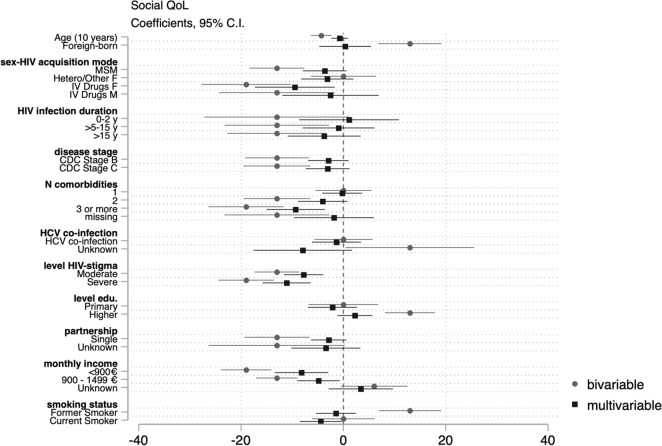
Coefficient plot: Bivariable and multivariable quartile regression analysis of social QoL.

Older PLWH, MSM, and women and men who inject(ed) drugs reported lower social QoL scores than heterosexual/other men. Those who had been living with HIV for more than five years, had experienced symptomatic HIV or AIDS, had more than two comorbidities, reported moderate or severe HIV-related stigma, were single, had lower net monthly income, or were smokers had significantly lower social QoL scores compared to reference groups in bivariable analyses. Those who were foreign-born, were university educated, or were former smokers had significantly higher social QoL scores. Those who reported perceiving moderate or severe HIV-related stigma, had more than three co-morbidities, and earned < 1500€/month had lower social QoL scores in the adjusted analysis. Differences between MSM and heterosexual/other men were confounded by disease-related factors, but men and women who inject(ed) drugs had significantly lower median social QoL scores after adjusting for disease-related, socioeconomic, and behavioral factors compared to heterosexual/other men (Fig. 3).

**Figure 4:**
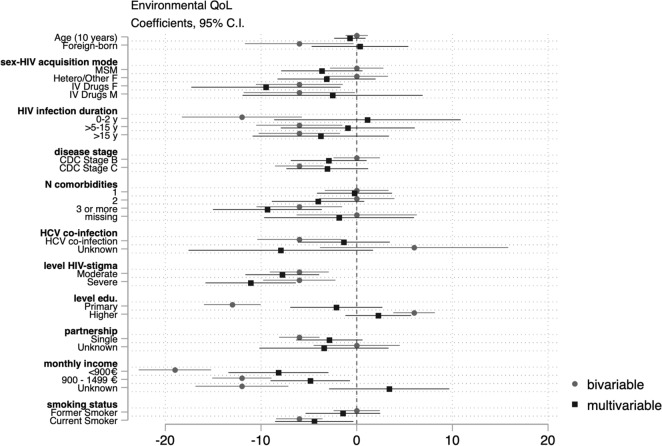
Coefficient plot: Bivariable and multivariable quartile regression analysis of environmental QoL.

Those who were foreign-born or had contracted HIV through IV drug use had poorer environmental QoL scores compared to reference groups in bivariable analyses. Those who had been diagnosed recently (0–2 years prior to the assessment) or for more than five years, had AIDS, had more than three comorbidities, had a history of HCV co-infection, reported moderate or severe HIV-related stigma, were less educated (primary versus secondary), earned < 1500€/month, or were single had lower median environmental QoL scores, whereas those who were university educated (university versus secondary) had higher environmental QoL scores in bivariable analyses. Current smokers also had lower environmental QoL scores than non-smokers. In the multivariable model, there was no evidence of a difference in the median environmental QoL according to birth place, sex-acquisition mode, duration of infection, or disease stage. However, differences, albeit smaller compared to other domains, persisted between those who had more than two comorbidities, reported moderate or severe HIV-related stigma, were single, and had less education or income (Fig. 4).

## Discussion

In Nouvelle Aquitaine, where the 90–90–90 UNAIDS targets were achieved in the late 2010s, ~ 63% of PLWH reported a good or very good QoL. Unfortunately, we are still far from achieving the ambitious 4th 90 or good QoL across the HIV care continuum. PLWH with ≥ 3 comorbidities, HIV-related stigma, or income of < 1500€/month had poorer median adjusted physical, psychological, social, and environmental QoL scores compared to reference groups.

Wilson and Cleary’s conceptual framework integrating biomedical and psychosocial models of health posits that the perception of GHS and overall HRQoL are distal outcomes of biological/physiological variables (e.g., immunological status). These biological and physical variables predict one’s symptom status, which influences one’s functional status or one’s ability to perform physical or social tasks, which affects one’s perception of GHS and ultimately QoL^15^. Several studies have examined the association between virological and immunological status and HRQoL with those with lower viral load measures and higher CD4 cell counts reporting better physical HRQoL in some^7,16^ but not all studies^17,18^. Our study, conducted five years into the universal treatment era, included very few PLWH with detectable viral loads (N = 53). The median physical QoL scores in PLWH with good virological response and those who were not completely virologically suppressed were not significantly different (69 vs. 63, *p* = 0.646). This discrepancy may be due to previous research being conducted at a time or in populations where a greater proportion of participants had detectable viral load measures or were immunologically compromised, thus increasing the statistical power to detect QoL differences at that time. Previous studies have also ascertained QoL using different instruments, such as the Short Form-36 or the MOS-HIV, which may be more sensitive to differences in physical health than the WHOQOL-BREF, which is designed to assess multidimensional QoL at a population level. Other studies have also found an association between immunological and virological status and psychological QoL^19,20^ but we have not. Past studies conducted prior to the universal treatment era have hypothesized that faster disease progression (or treatment failure) may have been distressing, resulting in poorer psychological QoL^19^. PLWH’s improved prognosis and access to well-tolerated treatments may alleviate psychological distress due to immunodeficiency.

More advanced disease stage and AIDS specifically have been associated with poorer physical QoL, but its effects on psychological, social and environmental QoL are less clear. Those who had experienced symptomatic HIV and AIDS were found to have poorer crude physical and psychological QoL and, to a lesser extent, poorer social and environmental QoL. However, these associations were mostly confounded by socio-economic and behavioural factors, speaking to the importance of social determinants of health in the universal treatment era.

Jia and colleagues have suggested that PLWH develop coping strategies over the course of their HIV infection, meaning those who have been diagnosed for years may actually have better QoL compared to those recently diagnosed^21^. We hypothesized that those diagnosed recently or having lived with HIV for more than five years would have poorer QoL than those diagnosed between two and five years, potentially reaping the benefits of an earlier diagnosis and response to ART as well as some time to process their diagnosis. Our results suggest that those who had been living with HIV for more than five years had poorer psychological QoL than those in the two to five year category in bivariable analyses, in contrast with Jia’s conclusions regarding PLWH’s ability to cope, but not in multivariable analyses, suggesting confounding by other factors. More nuanced analyses are required to better understand whether duration of infection truly translates to better psychological QoL via a coping mechanism.

As PLWH age, they are increasingly at risk of developing age-associated non-communicable diseases^22^. Rodriguez-Penney et al. assessed the burden of comorbidities using the Charlton Index, finding it to negatively affect physical QoL, especially in older PLWH^23^.This is consistent with our findings. While having one comorbidity did not appear to result in lower QoL domain scores compared to those with no comorbidities, we found that those with three or more comorbidities had significantly poorer physical QoL as well as psychological, social, and environmental QoL, whereas those with two comorbidities had significantly poorer psychological and environmental QoL, adjusting for other factors. Furthermore, as our measure of multi-morbidity is a count of the most common non-communicable diseases affecting PLWH, we may have underestimated its association with poorer multidimensional QoL. Had we adopted a broader definition, encompassing rarer comorbidities, the effect sizes may have been more pronounced^24^. This finding has major implications for HIV care in years to come as an estimated 28% of PLWH will have three or more non-communicable diseases in 2030, posing a threat not only their physical QoL but their psychological, social and environmental QoL as well ^22^.

Nearly one-third of participants reported experiencing either moderate or severe HIV-related stigma. Goffman defines stigma as “an attribute that is deeply discrediting, which reduces the bearer from a whole person to a tainted, discounted one”^25^. We found HIV-related stigma to be consistently associated with significantly lower median physical, psychological, social, and environmental QoL scores. Differences in QoL scores between those who reported HIV-related stigma and those who did not were much larger than those reported by heterosexual/other men who had experienced symptomatic HIV or AIDS, but not HIV-related stigma. This finding is consistent with previous research, showing HIV-related stigma to be strongly associated with poorer HRQoL, specifically in the psychological and social QoL^26,27^. Similar patterns have been observed in research on depressive syndrome in PLWH compared to those in the general population, wherein we observed a dose–response relationship between the prevalence of depressive syndrome and level of HIV-related stigma^28^. Different forms of HIV-related stigma, which are potentially compounded by intersectional stigma faced by populations disproportionately affected by HIV (e.g. sex workers, people who inject drugs or MSM), remain prevalent and represent a significant barrier to achieving good QoL in PLWH^29^. The strength and consistency of these associations should encourage investment in and evaluation of multi-level interventions designed to combat HIV-related and intersectional stigma experienced by PLWH.

Our analysis highlights the importance of social determinants of health in our understanding of which factors are associated with poorer multidimensional QoL. Higher socioeconomic status has been consistently associated with better HRQoL^30^. We have also found this to be true, with those with lower income, more so than education, reporting poorer QoL. Furthermore, the effect of several disease-related factors appears to be confounded by income, as these differences disappeared when restricted to those earning at least 1500€/month. These findings are in line with Ferrans et al.’s revisions to Wilson and Cleary’s conceptual framework integrating biomedical and psychological models of health, arguing that individual or environmental characteristics can influence biological and physical variables, symptoms and functional status, or the perception of GHS and QoL independently of disease status^31^. While France has a strong, but increasingly stressed, health system and an array of social services, they appear to have eased but not overcome an apparent social gradient in QoL, whereby people who are less advantaged have worse QoL compared to their more advantaged counterparts. Interventions aimed at improving PLWH’s health-related or, better yet, their multidimensional QoL must combine interventions targeting the individual with those addressing structural or contextual factors, which modify both risks and outcomes.

Smokers had poorer physical QoL scores compared to past and never-smokers, after adjusting for other factors, albeit not alcohol or drug misuse. These findings are in line with those of Crothers et al. who found that QoL scores, measured using the physical component summary of the SF-12, were 3.3 points lower in smokers after adjusting for race/ethnicity, CD4 cell count, HIV RNA level, hemoglobin, illegal drug, and alcohol use in HIV-positive veterans^32^. Interventions for smoking cessation, which take into account PLWH’s specificities, should continue to be prioritized.

### Strengths and limitations

Our study adds to the evidence regarding QoL in PLWH in the universal treatment era. The WHOQOL-BREF, an instrument designed to measure health and QoL as understood by the WHO, has provided a comprehensive and nuanced view of PLWH’s QoL of PLWH. Miners et al. measured HRQoL using the EQ-5D-3L, whereas Popping et al. and Kall et al. used the EQ-5D-5L in their studies comparing the HRQoL of PLWH with population norms^9,10,33^. Generic EuroQol instruments are brief, easy to administer, and generate a cardinal index of health for use in economic evaluations. The availability of population norms in several countries has heightened their appeal and facilitated their use. However, the EQ-5D, particularly the 3-level version, has been known to generate highly skewed scores with large ceiling effects^34^. Furthermore, the ED-5D covers mobility, self-care, usual activities, pain/discomfort and anxiety and depression, restricting its breadth to primarily physical and psychological QoL. We provide evidence of PLWH’s psychological, social, and environmental QoL, adding to the snapshot of their mean EQ-5D utility scores compared to the general population norms. However, our study has limitations. First, the study design may have resulted in the exclusion of foreign-born PLWH. Second, it may suffer from non-random sampling and nonresponse. However, comparisons with those engaged in regular care suggest that our sample is largely representative (Supplementary Material I).

## Conclusions

Our study underscores the need to address structural factors, such as PLWH’s socio-economic conditions, in addition to those indirectly related to living with HIV, namely multi-morbidity and stigma, to improve PLWH’s broader QoL. Redistributive policies and ongoing access to a high standard of care are needed to attain a good or excellent QoL in most PLWH.

### Supplementary Information

Supplementary Table 1.

## Data Availability

The datasets used and/or analyzed during the current study are available from the corresponding author upon reasonable request.
