# Supplementary material for: Factors associated with poorer quality of life in people living with HIV in southwestern France in 2018–2020 (ANRS CO3 AQUIVIH-NA cohort: QuAliV study)
Source: Sci Rep. 2023 Oct 2;13:16535. doi: 10.1038/s41598-023-43434-x (PMC10545822; doi:10.1038/s41598-023-43434-x)
Supplement: Supplementary file 1 — Supplementary Table 1. [file 41598_2023_43434_MOESM1_ESM.docx]

Table 1 : QuAliV sample and ANRS CO3 AQUIVIH-NA cohort participant characteristics (2018-2020)

|  |  | QuAliV Sample (N=965) | | ANRS CO3 AQUIVIH (N=4819) | |
| --- | --- | --- | --- | --- | --- |
|  |  | N *(%)* | Median  (Q1, Q3) | N (%) | Median  (Q1, Q3) |
| **Sex** | |  |  |  |  |
|  | Male | 726 *(75.3%)* |  | 3470 *(71.9%)* |  |
|  | Female | 239 *(24.7%)* |  | 1347 *(27.9%)* |  |
|  | Transgender | // |  | 2 *(0.04%)* |  |
| **Age (years)** | |  | 55 (48, 61) |  | 54 (45, 60) |
| **Place of birth** | |  |  |  |  |
|  | France | 832 (*86.2%)* |  | 3861 *(80.1%)* |  |
|  | Foreign | 133 *(13.8%)* |  | 958 *(19.9%)* |  |
| **HIV Transmission** | |  |  |  |  |
|  | MSM | 481 *(49.8%)* |  | 2087 *(43.3%)* |  |
|  | Heterosexual | 321 *(33.3%)* |  | 1846 *(38.3%)* |  |
|  | IV Drug Use | 102 *(10.6%)* |  | 525 *(10.9%)* |  |
|  | Other | 61 *(6.3%)* |  | 361 *(7.5%)* |  |
| **AIDS** | | 188 *(19.5%)* |  | 905 *(18.8%)* |  |
| **Duration of HIV Infection** | |  | 21 (12, 28) |  | 19 (10, 27) |
|  | *Missing^1^* | // |  | 1 *(0.02%)* |  |
| **Nadir CD4 cell count (/mm3)** | |  | 251 (131, 392) |  | 254 (134, 394) |
|  | *Missing^1^* | 11 *(1.2%)* |  | 50 (1.0%) |  |
| **Last viral load, <50 copies** | | 858 *(94.1%)* |  | 4194  *(92.0%)* |  |
|  | *Missing^1^* | 53 *(5.5%)* |  | 269 *(5.6%)* |  |
| **Last CD4 cell count (/mm3**) | |  | 692 (513, 911) |  | 697 (502, 910) |
|  | *Missing^1^* | 100 *(10.4%)* |  | 336 *(7.0%)* |  |
| **Last CD4/CD8** | |  | 0.98 (0.66, 1.4) |  | 0.94 (0.63, 1.4) |
|  | *Missing^1^* | 132 *(13.7%)* |  | 497 *(10.3%)* |  |
| **Number of comorbidities^2^** | |  |  |  |  |
|  | None | 438 *(45.4%)* |  | 2420 *(50.1%)* |  |
|  | 1 | 231 *(23.9%)* |  | 1106 *(22.9%)* |  |
|  | 2 | 145  *(15.0%)* |  | 602 *(12.5%)* |  |
|  | 3 | 104 *(19.8%)* |  | 452 *(9.4%)* |  |
|  | *Unknown* | 47 *(4.87%)* |  | 247 *(5.1%)* |  |
| **Past HCV infection** | | 182  *(18.9%)* |  | 934  *(20.2%)* |  |
|  | *Missing^1^* | 30 *(3.1%)* |  | 197 *(4.1%)* |  |
| **Past HBV infection** | | 67 *(6.9%)* |  | 296  *(6.6%)* |  |
|  | *Missing^1^* | 59 *(6.1%)* |  | 314 *(6.5%)* |  |

1 Percentages calculated excluding missing data. Missing data presented as number and percentage of missing data overall.

2 Including chronic renal failure, history of cardiovascular events, hypertension [taking antihypertensive treatment], diabetes, and cancer
